# Assessment of QuantuMDx Q-POC Assay for Rapid Detection of SARS-CoV-2 Using Middle Turbinate Swabs

**DOI:** 10.1128/spectrum.04256-22

**Published:** 2023-03-28

**Authors:** Mariana Fernandez-Pittol, Juan Carlos Hurtado, Maryam Ali, Álvar Simarro, Fadiana Proaño, Maria Sierra, Jordi Vila

**Affiliations:** a Department of Clinical Microbiology, Center for Biomedical Diagnosis (CDB), Hospital Clinic-Universitat de Barcelona, Barcelona, Spain; b Department of Basic Clinical Practice, School of Medicine, University of Barcelona, Barcelona, Spain; c Global Health Institute, ISGLOBAL, Barcelona, Spain; d CIBER Enfermedades Infecciosas (CIBERINFEC), ISCIII, Madrid, Spain; Taichung Veterans General Hospital

**Keywords:** evaluation, assay, RT-PCR, SARS-CoV-2

## Abstract

Currently, a rapid detection of SARS-CoV-2 in clinical settings such as patients from emergency surgery is needed. The QuantuMDx Q-POC assay is a real-time-PCR test that was created for the rapid detection of SARS-CoV-2 in only 30 min. This study aimed to compare QuantuMDx Q-POC with our standard algorithm with Cobas 6800 for SARS-CoV-2 detection. The samples were run in parallel in both platforms. First, a comparison analysis was carried out. Second, the limit of detection was determinate in both platforms using a serial dilution of SARS-CoV-2 inactivated virus. A total of 234 samples were analyzed. For a Ct <30, the sensitivity and specificity was 100.0% and 92.5%, respectively. Positive predictive value was 86.2% and negative predictive value was 100.0%. Both COBAS 6800 and QuantuMDx Q-POC could detect up to 100 copies/mL. The QuantuMDx Q-POC system it is a reliable option when a rapid detection of SARS-CoV-2 is necessary.

**IMPORTANCE** In different health care settings, such as patients from emergency surgery, rapid detection of SARS-CoV-2 is needed. The QuantuMDx Q-POC is an automatized fast workflow platform based on detection of three genes: two genes encoding structural proteins that can be used to differentiate SARS-CoV-2 from other coronavirus and a third target gene encoding a nonstructural region that is unique for SARS-CoV-2 such as the open reading frame (*ORF1*). This assay enables a rapid detection of SARS-CoV-2 with a high sensitivity in a short time frame (30 min). Therefore, QuantuMDx is a simple, rapid and easy SARS-CoV-2 detection test from direct middle nasal swabs.

## INTRODUCTION

The pandemic of coronavirus disease 2019 (COVID-19) caused by the severe acute respiratory syndrome coronavirus 2 (SARS-CoV-2) (1) has infected as of today 650,332,899 individuals and claimed millions of lives across the globe according to the World Health Organization (WHO) data on December 13, 2022 (2). The clinical presentation of COVID-19 disease is different from patient to patient. However, the most common symptoms include fever, fatigue, cough, expectoration, sputum production, and anorexia (3). Different studies also described that a proportion of patients may be asymptomatically infected with SARS-CoV-2 (4). In general, the management of these patients requires an early diagnosis, isolation, and measures to prevent the infection (4). During the beginning of the pandemic, it was necessary to test the maximum number of patients to try to cut the chain of infections. At this time, the need is focused on some epidemiological and clinical situations, such as rapid and effective contact tracing, surveillance at different levels, the implementation of infection prevention, and control measures at the local or regional level and contributing to the proper care of patients. Several commercial COVID-19 tests have been developed for the detection of SARS-CoV-2 (5, 6). Most of these tests are based on the real-time reverse transcription-PCR (RT-PCR) methods (7). This technique remains as the gold standard technique for the diagnosis of COVID-19 (8). In inpatient care sites, there are different needs to have the results; this has led to the development of different RT-PCR protocols and platforms. The QuantuMDx Q-POC assay (QuantuMDx, UK) based on RT-PCR was created for a rapid detection of SARS-CoV-2, targeting three loci: two structural proteins that can be used to differentiate SARS-CoV-2 from other coronavirus as the spike protein (*S* gene) and nucleocapsid protein (*N* gene). Finally, a third target a nonstructural region that is unique for SARS-CoV-2, such as the open reading frame (*ORF1*). This rapid system allows to obtained results in just 30 min (9). In general, these types of tests can be applied in situations described as relevant cases where a patient needed an urgent result. Therefore, we can use this strategy in managing patients in the emergency room, for emergency surgeries, lifesaving radiological interventions, intensive care unit (ICU) patients, or health care workers.

Another approach in COVID-19 diagnostics is using automated systems that can simultaneously process many samples at the same time. The Cobas 6800 (Roche molecular system, Branchburg, NJ) is one of them. This system allows qualitative detection of SARS-CoV-2 through two target regions: *ORF1* a/b (specific for SAR-CoV-2) and the detection of pan-Sarbecovirus (through a conserved region in the structural protein of the envelope gene *E*) for the *Sarbecovirus* subgenus that includes SARS-CoV-2. Disadvantages of this diagnostic pathway are that trained staff are required, results are not available in real-time, and this platform cannot be used in a point-of-care (POC); it must be located within a microbiology laboratory or central core laboratory. Therefore, this cannot be considered a rapid detection assay (10). This study aimed to compare the sensitivity and specificity of QuantuMDx Q-POC assay with our standard routine algorithm with Cobas 6800 for SARS-CoV-2 diagnostic.

## RESULTS

**FIG 1:**
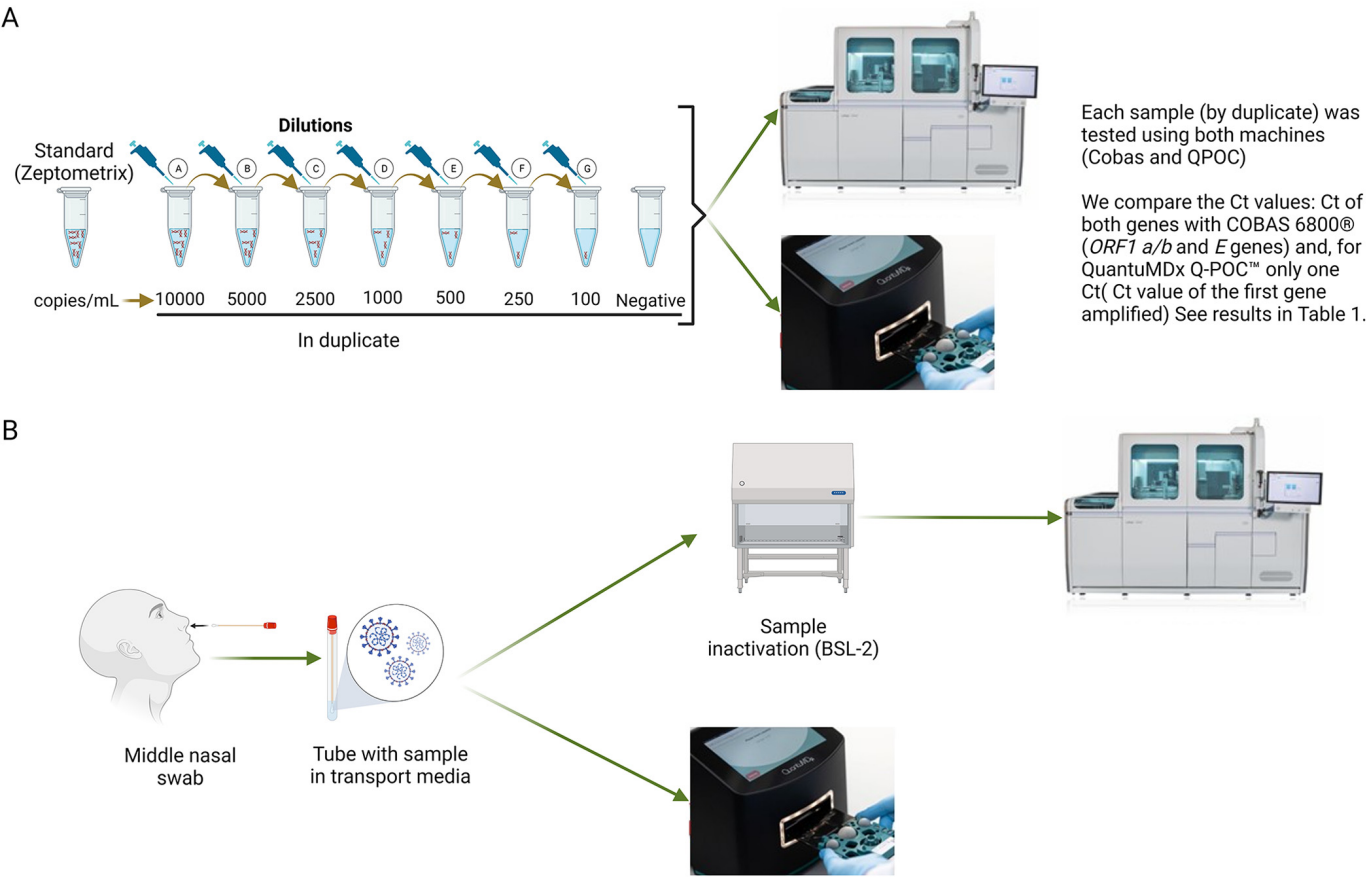
Comparison of the load viral and the processing of both platforms. (A) Comparison of the load viral of both platforms using a serial dilution of Zeptometrix SARS-CoV-2 inactivated virus standard. (B) The processing of samples using both platforms. Ct: Cycle threshold. BSL-2, Biosafety level 2. *NA, not applied. The Ct values of COBAS 6800 was expressed in scientific notation used one decimal.

The processing of both platforms and the data obtained is summarized in Fig. 1. Panel A showed viral load dilution method and panel B the workflow protocol since sampling. In Table 1, we can find the results of the serial viral dilution. Both COBAS 6800 and QuantuMDx Q-POC could detect up to 100 copies/mL.

**TABLE 1:** Dilutions results of zeptometrix SARS-CoV-2 amplification obtained with COBAS 6800 and QuantuMDx Q-POC

Sample ID	Serial dilution (copies/mL)	QuantuMDx Q-POC result	QuantuMDx Q-POC Ct value	COBAS 6800 result^*b*^	Cobas: ORF1 gene Ct value	Cobas: E gene Ct value
SARS-A	10,000	Positive	35.8	Positive	30.2	31.0
SARS-B	5,000	Positive	38.9	Positive	31.1	31.8
SARS-C	2,500	Positive	40.6	Positive	32.1	33.0
SARS-D	1,000	Positive	40.7	Positive	33.1	34.3
SARS-E	500	Positive	41.2	Positive	33.9	34.8
SARS-F	250	Positive	42.8	Positive	34.7	36.4
SARS-G	100	Positive	44.3	Positive	35.0	36.8
Negative control	NA^*a*^	Negative	0.0	Negative	0.0	0.0

a NA, not applied.

b The Ct values of COBAS 6800 was expressed in scientific notation used one decimal.

**FIG 2:**
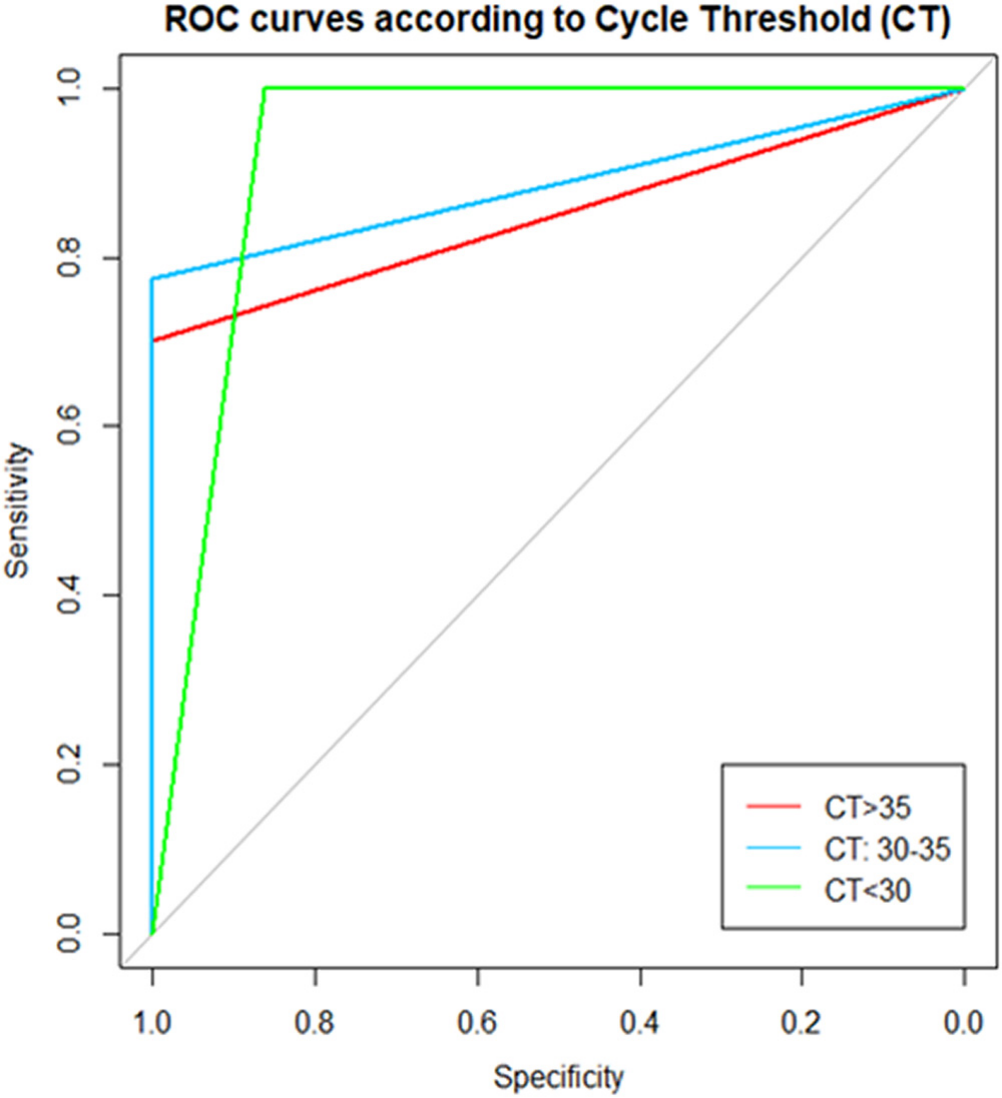
ROC curve considering the three different groups of thresholds for Ct.

A total of 234 samples were analyzed. A total of 147 were negative samples and 87 were positive using COBAS 6800. The prevalence of our data was 37.2%. Fig. 2 showed the ROC curve according to QuantuMDx Q-POC results of the different Ct values groups. For a Ct <30, the sensitivity (95% CI) and specificity (95% CI) was 100.0% (95.2 to 100.0) and 92.5% (87.2 to 96.0), respectively, with area under the curve (AUC) of 0.93 (0.89 to 0.97). Positive predictive value was 86.2% (77.1 to 92.7) and negative predictive value was 100.0% (97.5 to 100.0). The second group with Ct 30 to 35 was presented a sensitivity (95% CI) and specificity (95% CI) of 72.5% (63.6 to 80.3) and 100.0% (96.8 to 100.0), respectively, with an AUC of 0.89 (0.85 to 0.92). Positive predictive value was 100.0% (95.8 to 100.0) and negative predictive value was 77.6% (69.9 to 84.0). Finally, for the third group Ct ≥35 the sensitivity (95% CI) and specificity (95% CI) was 66.4% (57.6 to 74.4) and 100.0% (96.5 to 100.0), respectively, with AUC of 0.85 (0.81 to 0.89). Positive predictive value was 100.0% (95.8. to 100.0) and negative predictive value was 70.1% (62.0 to 77.3). Table 2 summarized all the results.

**TABLE 2:** Statistical results of Q-POC assay according to cycle threshold (CT) value

Cycle threshold (Ct)	TRUE positive (n)	TRUE negative (n)	False positive (n)	False negative (n)	Sensitivity (95% CI)	Specificity (95% CI)	PPV^*a*^ (95% CI)	NPV^*a*^ (95% CI)
>35	87^*b*^	103	0	44	66.4	100.0	100.0	70.1
					(57.6 to 74.4)	(96.5 to 100.0)	(95.8 to 100.0)	(62.0 to 77.3)
30 to 35	87	114	0	33	72.5	100.0	100.0	77.6
					(63.6 to 80.3)	(96.8 to 100.0)	(95.8 to 100.0)	(69.9 to 84.0)
<30	75	147	12	0	100.0	92.5	86.2	100.0
					(95.2 to 100.0)	(87.2 to 96.0)	(77.1 to 92.7)	(97.5 to 100.0)

a PPV predictive positive value; NPV, negative predictive value.

b The number of samples analyzed was the same in each group.

## DISCUSSION

Rapid identification of SARS-CoV-2 in some clinical situations is needed. Since the beginning of the pandemic, the U.S. Food Drug Administration granted emergency authorizations to qualitative test of SARS-CoV-2 in clinical samples (11). We can find several rapid RT-PCR commercial tests in the market that allow to obtain results between 15 to 60 min, depending on the platform used. In general, for these techniques, we can amplify one or two unique regions for SARS-CoV-2 and one region that it is shared for all coronavirus types (6). To date, some studies have been published on the performance of these rapid tests and the positive percent agreement that is ranging among 68% to 100% (12). The QuantuMDx Q-POC is an automatized fast workflow platform which enables a rapid detection of SARS-CoV-2 in a short time frame from directed middle nasal (MN) swabs (9). In general, the software is very easy to use and the report is clear to interpretate without indeterminate results, reporting only positive, negative, or invalid results. Compared to antigen-based POC tests, which can take 15 to 30 min, QuantuMDx Q-POC has a similar response time or just a few extra minutes (for positive samples, response time can be 20 min), and unlike of antigen tests, it can provide a Ct value. In a recent meta-analysis, the authors found that molecular tests had better sensitivity; however, in the future a control quality analysis of rapid molecular platforms in view of the progress of SARS-CoV-2 variants will be necessary (13). In this analysis, compared with a robust platform, QuantuMDx Q-POC showed a sensitivity and specify of 100.0% and 92.5%, respectively, in the group of patients with positive results and a Ct value <30. These data are similar to previous report of QuantuMDx Q-POC that showed an overall sensitivity of 96.9% in cases with a Ct value ≤ 35 (10).

Nevertheless, differences observed in Ct values were COBAS 6800 showed better Ct results in both genes in comparison with QuantuMDx Q-POC values. In addition, this difference was also observed in viral load dilution calculation, as the only first two dilutions presented a Ct value <40; however, both systems were able to detect until 100 copies/mL of the load viral concentration. It may be that this difference between platforms occurs in some cases due to the characteristic of the sample, inadequate sample collection, or the beginning of the infection where patients could present a low-level of positivity and report as a falsely negative test (14). On the contrary, a false positivity result is also possible. This was previously reported in RT-PCR tests that were able to detect genes with high Ct value. In these cases, the hypothesis of the low positivity level can possibly be related to a late stage of infection. In general, the use of Ct values has been a controversial issue since the beginning of the pandemic. Although Spain’s recommendation established that a case with Ct ≥30 to 35 can be considered not an infective case, it is not so clear that Ct values can be a suitable tool to discriminate infectives cases. This remains an unsolved issue.

Finally, we can conclude that QuantuMDx Q-POC system it is a reliable option for rapid a detection of SARS-CoV-2 compared with a robust platform such as COBAS 6800.

## MATERIALS AND METHODS

Middle nasal (MN) swabs were consecutively included during the fourth wave in Spain (March 15, 2021 to June 19, 2021) from a third-level hospital. First, all the samples were run in parallel and were collected in a 3 mL of MSwab sample collection, transport, and preservation medium (Copan Diagnostics, Italy). For our lab routine: 400 μL of the sample was inactivated for processing in COBAS 6800. Rapid test-QuantuMDx Q-POC: direct sample was used. Briefly, for processing, this assay media tube with the specimen collection was rapidly mixed inverting the tube several times. Then, 400 μL of MN was transferred to the sample chamber assay cassette. We compared the load viral of both platforms using a serial dilution of Zeptometrix SARS-CoV-2 inactivated virus (wild-type virus. Strain USA-WA1/2020) having as a lower detection limited 100 copies/mL. The serial dilution was processed in duplicate and a negative control was included. The inactivated virus was diluted in medium viral swab (Copan Diagnostics, Italy) and each dilution tube contained 500 c/mL RNaseP plasmid. The concentration of SARS-CoV-2 was 1.08× 10^6^. Finally, we compare the Ct values: Ct of both genes with COBAS 6800 (*ORF1 a/b* and *E* genes) and, for QuantuMDx Q-POC only one Ct value, because the platform has the ability to display only the Ct value of the first gene amplified, but it is unable to specify at which gene belong to. During all the process, a protocol to avoid cross-contamination was followed. First, all the samples were processed in a laminar flow cabin (bio security cabin level 2). A previous step of inactivation was used for COBAS 6800 and, in all the cases before transfer, the samples out of the cabin were inactivated or not; all the tubes were decontaminated individually with bleach at 0.5%.

Moreover, we compared all the results obtained from both platforms. In our study, the positive results were analyzed in three groups: first positives with a cycle threshold (Ct) <30, between 30 and 35 and ≥35. However, following national guidelines recommendation patients of the last two groups were considered a positive not infectives cases (15). For the comparison analyses, sensitivity, specificity, positive predictive value, and negative predictive value was calculated using COBAS 6800 as a reference standard technique. Also, the area under the receiver operating characteristic curve (AUC-ROC) of the predictions of the model to assess measures of sensitivity and specificity in the three established groups to evaluate the performance of QuantuMDx Q-POC was calculated. The analysis was performed using Stata, version 16 (TX, USA).

The study was approved by the local ethical committee (HCB/2020/1001).
