# Erratum for Fernandez-Pittol et al., “Assessment of QuantuMDx Q-POC Assay for Rapid Detection of SARS-CoV-2 Using Middle Turbinate Swabs”

**DOI:** 10.1128/spectrum.03249-23

**Published:** 2023-10-26

**Authors:** Mariana Fernandez-Pittol, Juan Carlos Hurtado, Maryam Ali, Álvar Simarro, Fadiana Prodaño, Maria Sierra, Jordi Vila

## ERRATUM

Volume 11, no. 2, e04256-22, 2023, https://doi.org/10.1128/spectrum.04256-22.

Page 2, sentence beginning “This study aimed to compare”: “diagnostic” should be “diagnosis.”

Page 3, Fig. 1A: In the first sentence of text in this panel, “QPOC” should be “Q-POC.”

Page 3: The sentence beginning “Nevertheless” should read, “Nevertheless, differences were observed in Ct values, and COBAS 6800 showed better Ct results in both genes in comparison with QuantuMDx Q-POC values.”

Page 4, sentence beginning “Although Spain’s recommendation”: “infectives cases” should be “infective cases.”

Page 4: The sentence beginning “Finally” should read, “Finally, we can conclude that the QuantuMDx Q-POC system is a reliable option for rapid detection of SARS-CoV-2 compared with a robust platform such as COBAS 6800.”

Page 5, sentence beginning “The inactivated virus”: “medium viral swab” should be “viral swab medium.”

Page 5, sentence beginning “However, following national guidelines”: “not infective” should be “noninfectious.”

These corrections do not change the conclusions of the paper.

